# Optimal Low-Flow Time of Extracorporeal Cardiopulmonary Resuscitation for Favorable Neurological Outcomes: A Risk-Stratified Approach

**DOI:** 10.3390/jcm15072541

**Published:** 2026-03-26

**Authors:** Hyo Seok Oh, Joonghyun Ahn, Ryoung-Eun Ko, Jeong Hoon Yang, Yang Hyun Cho, Jeong-Am Ryu

**Affiliations:** 1 Department of Critical Care Medicine, Samsung Medical Center, Sungkyunkwan University School of Medicine, Seoul 06351, Republic of Korea; hyoseok.oh2101@gmail.com (H.S.O.); ryoungeun.ko@samsung.com (R.-E.K.); jhysmc@gmail.com (J.H.Y.); 2 Biomedical Statistics Center, Data Science Research Institute, Samsung Medical Center, Seoul 06351, Republic of Korea; jhguy.ahn@samsung.com; 3 Division of Cardiology, Department of Medicine, Samsung Medical Center, Sungkyunkwan University School of Medicine, Seoul 06351, Republic of Korea; 4 Department of Thoracic and Cardiovascular Surgery, Samsung Medical Center, Sungkyunkwan University School of Medicine, Seoul 06351, Republic of Korea; yanghyun.cho@samsung.com; 5 Department of Neurosurgery, Samsung Medical Center, Sungkyunkwan University School of Medicine, Seoul 06351, Republic of Korea

**Keywords:** extracorporeal membrane oxygenation, heart arrest, cardiopulmonary resuscitation, risk assessment, treatment outcome

## Abstract

**Background**: Determining the optimal duration of extracorporeal cardiopulmonary resuscitation (ECPR) remains challenging, as patient outcomes may vary significantly based on individual characteristics. We aimed to establish critical time thresholds for achieving favorable neurological outcomes with ECPR across different risk groups, potentially providing more tailored guidance for clinical decision-making. **Methods**: This single-center retrospective study screened 279 adult patients who received ECPR between 2013 and 2020. Through multivariate analysis of various clinical parameters, we developed a pragmatic bedside risk stratification framework to identify groups with different prognostic profiles. The primary outcome was neurological status at discharge, assessed by the Cerebral Performance Categories scale. **Results**: In multivariate analysis, age greater than 50 years with asystole (adjusted odds ratio [OR]: 4.89, 95% confidence interval [CI]: 1.41–17.00) or pulseless electrical activity (adjusted OR: 9.70, 95% CI: 2.80–33.60), aspartate transaminase (adjusted OR: 1.52, 95% CI: 1.15–1.99), creatinine (adjusted OR: 2.08, 95% CI: 1.30–3.34), initial lactate (adjusted OR: 1.88, 95% CI: 1.27–3.45), and low-flow time (adjusted OR: 3.50, 95% CI: 2.02–6.06) were associated with poor neurological outcomes. Based on these findings, we identified three distinct risk groups showing different acceptable low-flow time thresholds: low-risk (38 min), moderate-risk (27 min), and high-risk (20 min). Notably, no favorable neurological outcomes were observed beyond 70 min in the low-risk group and 90 min in moderate/high-risk groups. Risk group stratification effectively predicted neurological outcomes across different low-flow time intervals. **Conclusions**: Risk-stratified evaluation of low-flow time (cardiac arrest to ECMO pump-on) provides clinically relevant thresholds for different patient groups, suggesting that continuation of ECPR may be warranted in low-risk patients even with extended low-flow times. This approach may enable more personalized decision-making in ECPR implementation.

## 1. Background

Extracorporeal cardiopulmonary resuscitation (ECPR), utilizing extracorporeal membrane oxygenation (ECMO) during cardiac arrest, has shown promise for patients unresponsive to conventional cardiopulmonary resuscitation (CPR). Recent systematic reviews have demonstrated improved survival rates and neurological outcomes with ECPR compared to conventional CPR in selected populations, leading to increased implementation worldwide [1,2].

However, determining the optimal duration of CPR remains one of the most challenging decisions clinicians face during cardiac arrest. While ECPR using ECMO may allow for extended resuscitation times compared to conventional CPR, establishing the optimal duration continues to be a significant challenge. The impact of prolonged CPR varies among patient populations, with younger patients showing greater physiological reserve and resilience. Additionally, initial shockable rhythms such as ventricular fibrillation or pulseless ventricular tachycardia have been associated with improved outcomes in conventional CPR, suggesting their potential significance in ECPR success [3,4]. These patient characteristics may translate into differences in tolerable low-flow times. For instance, a young patient with a shockable rhythm may have substantially different hypoxia tolerance and recovery potential compared to an elderly patient with asystole. This variability in patient characteristics and their potential impact on outcomes strongly suggests the need for differentiated low-flow time criteria based on patient-specific factors [5,6].

Despite the apparent importance of patient-specific characteristics in ECPR outcomes, most current studies propose uniform duration criteria for all patients [7,8]. To address this gap, our single-center retrospective study aims to investigate the optimal duration of ECPR according to risk groups for favorable neurological outcomes. Unlike previous studies that analyzed optimal low-flow time of ECPR in overall patient populations, our study examines this relationship by stratifying patients into different risk groups. Through this approach, we aim to establish critical time thresholds for achieving favorable neurological outcomes with ECPR across different risk groups, potentially providing more tailored guidance for clinical decision-making regarding low-flow time.

## 2. Methods

### 2.1. Study Population

**Figure 1:**
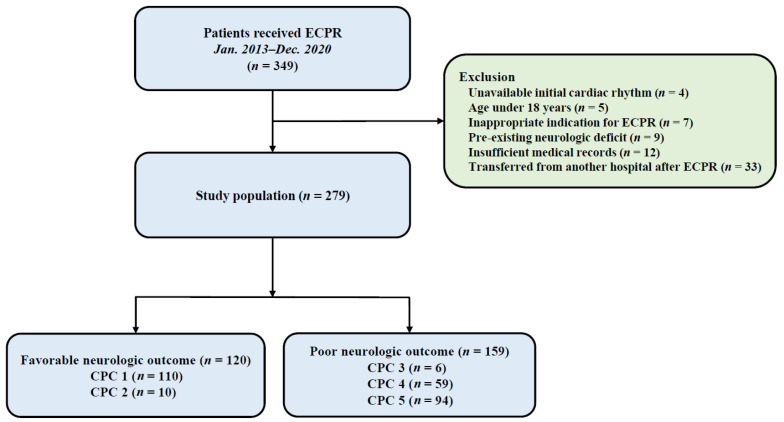
Study flow chart. ECPR, extracorporeal cardiopulmonary resuscitation; CPC, Cerebral Performance Categories scale.

This investigation constitutes a retrospective, observational study conducted at a single center. It encompasses a cohort of adult patients who received ECPR during hospitalization from January 2013 through December 2020. The Samsung Medical Center’s Institutional Review Board (IRB) sanctioned this study (IRB No. 2023-05-052-001). Due to the study’s retrospective nature, the requirement for informed consent was waived by the IRB. The cohort comprised all successive patients subjected to ECPR within the specified timeframe, totaling 283 individuals. Of these, 4 patients were excluded due to unavailable initial cardiac rhythm data, as initial rhythm is a primary stratification variable in our analysis. The study also excluded patients under the age of 18, candidates with contraindications for ECPR, patients with pre-existing serious neurological impairments (including traumatic brain injury, significant cerebrovascular accidents, malignant cerebral neoplasms, or advanced dementia), cases with inadequate medical documentation, and individuals transferred post-ECPR from alternative institutions (Figure 1).

### 2.2. Definitions and Outcomes

In the context of this study, we conducted a retrospective analysis of patient data, which included baseline characteristics, comorbidities, behavioral risk factors, intensive care management strategies, and laboratory results. These data were sourced from our institution’s dedicated ‘Clinical Data Warehouse Darwin-C,’ a repository designed to enable researchers to query and access anonymized medical records from electronic archives efficiently. Within this study, ‘ECPR’ was delineated as the application of venoarterial ECMO in response to cardiac arrest, with the duration from cardiac arrest to ECMO initiation (arrest to ECMO pump-on time) explicitly measured [9]. The decision to initiate ECPR was made by the attending physician according to our institution’s protocol, which follows current international guidelines [10,11]. The ECPR procedure was performed by the ECMO team at our institution, which consists of cardiovascular surgeons, interventional cardiologists, and ECMO specialists available 24/7. Venoarterial ECMO was established using either the Capiox Emergency Bypass System (Terumo Corporation, Tokyo, Japan) or the HLS Set with Rotaflow centrifugal pump (Maquet Cardiopulmonary AG, Rastatt, Germany). Shockable rhythm was defined as the presence of either ventricular tachycardia (VT) or ventricular fibrillation (VF) on the first monitored cardiac rhythm during the cardiac arrest event. Non-shockable rhythms included asystole and pulseless electrical activity. In this study, we adopted standardized time definitions consistent with the Utstein reporting guidelines. Low-flow time was defined as the interval from cardiac arrest to ECMO pump-on, encompassing the entire period of conventional CPR and cannulation. For in-hospital cardiac arrest (IHCA) patients, the arrest time was the time of witnessed cardiac arrest documented by the attending medical team. For out-of-hospital cardiac arrest (OHCA) patients, the arrest time was defined as the emergency medical service (EMS) dispatch time. The primary time variable was low-flow time, representing the total ischemic burden prior to establishment of extracorporeal circulatory support. The primary endpoint was the neurological status at the point of hospital discharge, quantified by the Glasgow-Pittsburgh Cerebral Performance Categories (CPC) scale [12]. Scores on the CPC scale range from 1 (good cerebral performance) to 5 (brain death), with scores of 1 and 2 indicative of favorable neurological outcomes and scores of 3 to 5 indicative of poor neurological outcomes [13,14]. Patient medical records were meticulously evaluated, and CPC scores were assigned upon consensus between two authors (JAR and HSO). In cases of disagreement, a third reviewer was consulted to reach a final decision. Inter-rater discrepancies occurred in only 5 of 279 cases (1.8%), all involving distinctions within the unfavorable outcome category (CPC 3 vs. CPC 4), with no disagreements crossing the favorable (CPC 1–2) versus unfavorable (CPC 3–5) boundary.

### 2.3. Statistical Analyses

Continuous variables are presented as means accompanied by standard deviations. Categorical variables are represented as numbers with subsequent percentages. Comparative analysis of data employed Student’s *t*-test for continuous variables and the Chi-square test for categorical counterparts. It should be noted that our analytical approach was designed as a pragmatic bedside risk stratification framework rather than a formally optimized predictive model. The primary objective was to develop a clinically applicable tool using variables immediately available at the time of the ECPR decision. To identify independent predictors of neurological outcomes, we performed multiple logistic regression analysis, including clinically relevant variables: age, sex, comorbidities, habitual risk factors, and ECPR-specific factors such as arrest subtypes, ECMO complications, and ICU management strategies. Based on the multivariate analysis results, we identified key prognostic factors for poor neurological outcomes. Subsequently, we developed several candidate risk-stratification models using combinations of these identified prognostic factors. The objective of this model is to optimize the determination of the most suitable low-flow time of ECPR, aiming to enhance patient outcomes. Additionally, this model facilitated the stratification of patients into three-tiered risk groups, enabling an in-depth investigation into the relationship between the duration of ECPR and the neurologic prognosis of the patients. The cumulative probability of favorable neurological outcomes at discharge was calculated for all eligible participants stratified by significant variables [15,16,17,18,19]. Low-flow time of ECPR would achieve a cumulative probability exceeding 99% of those with favorable neurological outcomes at discharge by three-tiered risk groups. Age-based stratification was determined through exploratory threshold characterization using restricted cubic splines, which identified a nonlinear relationship with a clinically meaningful inflection point at approximately 50 years. Low-flow time thresholds within each risk group were characterized by tabulating observed favorable outcome rates across 10 min intervals. For reference, we also report the low-flow time at which the combined sensitivity and specificity was highest in each risk group by using the Youden index [20,21]. All tests were two-sided, and *p*-values of less than 0.05 were considered statistically significant. For the primary stratification variables, there were no missing data for age; initial cardiac rhythm was unavailable for 4 patients (1.4%), who were excluded. For laboratory variables, missing data were handled using complete case analysis (AST: 16 patients [5.7%]; creatinine: 15 patients [5.4%]; complete cases: 247/279 [88.5%]). Sensitivity analyses using four imputation methods yielded consistent AUC values (0.800–0.803). Bootstrap internal validation yielded an optimism-corrected C-statistic of 0.775 (apparent C = 0.805; leave-group-out cross-validation C = 0.782), indicating minimal overfitting. Statistical analyses were performed with R Statistical Software (version 4.2.0; R Foundation for Statistical Computing, Vienna, Austria).

## 3. Results

### 3.1. Baseline Characteristics and Clinical Outcomes

Within the duration of this study, a total of 279 patients were included in the analysis, of which 120 (43.0%) achieved favorable neurological outcomes, while 159 (57.0%) did not. Patient demographics and clinical features are detailed in Table 1. Analysis of age, sex, and comorbidities revealed no significant differences between the groups, except for chronic kidney disease. Notably, the group with favorable neurological outcomes was characterized by a higher incidence of in-hospital cardiac arrests, the presence of shockable rhythms, initiation of ECPR in the coronary catheterization laboratory, and cardiac arrests attributable to ischemic heart disease and cardiac causes. Furthermore, this group was less likely to require continuous renal replacement therapy compared to the group with poor neurological outcomes. Notably, low-flow time was longer in the poor neurological group compared with the favorable neurological outcome (39.5 ± 22.8 min vs. 27.1 ± 18.1, *p* < 0.001). In laboratory tests, hemoglobin was lower in the poor neurological group than the favorable neurological group (9.3 ± 3.1 g/dL vs. 10.5 ± 2.6 g/dL, *p* = 0.001), but troponin I showed no significant difference between the two groups (24.9 ± 71.0 ng/mL vs. 18.2 ± 69.5 ng/mL, *p* = 0.502). Aspartate transaminase (311.5 ± 804.6 U/L vs. 145.7 ± 341.4 U/L, *p* = 0.025), blood urea nitrogen (28.3 ± 19.2 mg/dL vs. 20.3 ± 13.6 mg/dL, *p* < 0.001), creatinine (2.1 ± 2.2 mg/dL vs. 1.4 ± 1.3 mg/dL, *p* = 0.001), and initial lactate (12.5 ± 4.7 mmol/L vs. 9.5 ± 5.2 mmol/L, *p* < 0.001) were higher in the poor neurologic outcome group than in the favorable group (Table 1).

In the multivariate analysis, several factors were significantly associated with poor neurological outcomes (Table 2).

In the multivariate analysis, age greater than 50 years combined with specific cardiac rhythms showed significant associations with poor neurological outcomes: when combined with asystole (adjusted odds ratio [OR]: 4.89; 95% confidence interval [CI]: 1.41–17.00) and with pulseless electrical activity (adjusted OR: 9.70; 95% CI: 2.80–33.60). Similarly, elevated aspartate transaminase levels (adjusted OR: 1.52; 95% CI: 1.15–1.99), creatinine levels (adjusted OR: 2.08; 95% CI: 1.30–3.34), initial lactate levels (adjusted OR: 1.88; 95% CI: 1.27–3.45), and longer low-flow time (adjusted OR: 3.50; 95% CI: 2.02–6.06) were associated with poor neurological outcomes. These variables were subsequently incorporated into developing our risk stratification model.

### 3.2. Cumulative Probability and Cutoff of Low-Flow Time of ECPR for Favorable Neurological Outcomes at Discharge

**Figure 2:**
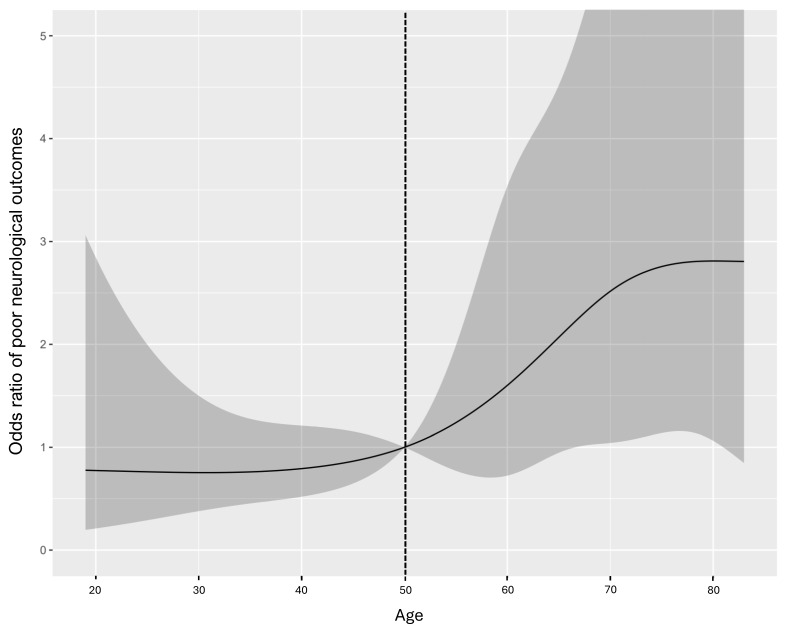
Age-dependent probability of poor neurological outcomes after extracorporeal cardiopulmonary resuscitation. The solid line represents the predicted probability of poor neurological outcomes based on patient age, with the shaded area indicating the 95% confidence interval. A marked increase in the probability of poor outcomes is observed around age 50 years, which was subsequently used as a cutoff point for risk stratification. The predicted probabilities were derived from a multivariate logistic regression model adjusted for low-flow time of extracorporeal cardiopulmonary resuscitation, aspartate transaminase, initial lactate levels, and first monitored rhythm. The vertical dotted line at age 50 represents the identified threshold for risk stratification.

The association between patient age and the probability of poor neurological outcomes was explored using restricted cubic splines, revealing a nonlinear relationship with a marked increase in risk beginning at approximately 50 years (Figure 2). This threshold was further corroborated by 10-year interval odds ratios.

**Figure 3:**
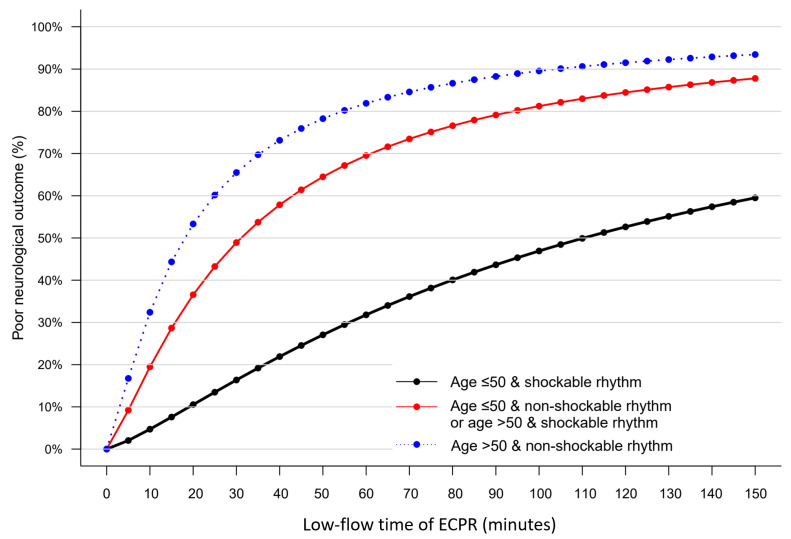
Relationship between the Low-flow time and probability of poor neurological outcomes stratified by risk group. Risk categories are defined as follows: low-risk patients with shockable rhythms and age under 50 years; moderate-risk patients with shockable rhythms and age 50 years or older, or patients with non-shockable rhythms and age under 50 years; high-risk patients with non-shockable rhythms and age 50 years or older.

Among the significant predictors identified in multivariate analysis, age and initial rhythm were selected as the primary stratification factors based on their immediate availability, established pathophysiological significance, and supporting evidence from prior studies. These two variables offer the critical advantage of being known instantaneously at the ECPR decision point, unlike laboratory markers requiring blood sampling and processing time. Subsequently, we established three risk categories: low-risk includes patients younger than 50 years with shockable rhythms; moderate-risk encompasses patients 50 years or older with shockable rhythms, or those younger than 50 with non-shockable rhythms; high-risk comprises patients 50 years or older with non-shockable rhythms. Figure 3 illustrates the relationship between low-flow time and the probability of poor neurological outcomes, stratified by these risk categories, demonstrating a stepwise increase in poor neurological outcomes from low to high-risk groups across all low-flow times of ECPR. This stratification effectively distinguished patients’ outcomes, with the low-risk group showing consistently better outcomes compared to moderate and high-risk groups.

Table 3 presents the cumulative proportion of patients achieving favorable neurological outcomes across different low-flow time intervals, stratified by risk groups. In the low-risk group, favorable outcomes continued to accumulate until 70 min of low-flow time, at which point all potential recoveries had occurred (100% of favorable outcomes achieved). For both moderate and high-risk groups, this accumulation continued until 90 min, after which no additional patients achieved favorable outcomes.

For reference, Table 4 presents the low-flow time at which the highest combined sensitivity and specificity was observed in each risk group using the Youden index: 38 min for the low-risk group, 27 min for the moderate-risk group, and 20 min for the high-risk group. These values are provided as supplementary reference points rather than definitive clinical thresholds, as our primary analytical approach—the cumulative outcome distribution presented in Table 3—offers clinicians a more flexible, context-dependent framework for decision-making. Notably, no favorable neurological outcomes were observed beyond 70 min of low-flow time in the low-risk group and beyond 90 min in the moderate and high-risk groups.

## 4. Discussion

In this single-center retrospective study, we investigated the optimal duration of ECPR for favorable neurological outcomes through risk stratification of patients. Our analysis revealed several key findings. First, based on the multivariate analysis, which identified age, AST, creatinine, initial lactate, initial rhythm, and low-flow time as significant predictors, we developed a risk stratification model that predominantly incorporated age and initial rhythm, as these were the most clinically applicable factors. This model effectively categorized patients into three risk groups with distinct outcomes. Our findings suggest that the prognostic impact of low-flow time is not uniform across patients but is modified by baseline clinical risk, supporting a risk-stratified rather than universal time threshold for ECPR decision-making. The acceptable low-flow time varied significantly among these groups: up to 38 min for the low-risk group (younger patients with shockable rhythms), up to 27 min for the moderate-risk group (older patients with shockable rhythms or younger patients with non-shockable rhythms), and up to 20 min for the high-risk group (older patients with non-shockable rhythms). More importantly, we found that favorable neurological outcomes could still be achieved beyond these optimal cutoff times, but with diminishing probability. However, no patients achieved favorable neurological outcomes when low-flow time exceeded 70 min in the low-risk group and 90 min in both moderate and high-risk groups, establishing these durations as the absolute time thresholds for ECPR continuation.

The distinctive aspect of our study lies in its risk-stratified approach to determining optimal low-flow time thresholds for ECPR. Previous studies have primarily focused on identifying a single optimal cutoff time for the entire patient population, thereby oversimplifying the complex relationship between ECPR timing and outcomes. Although our multivariate analysis identified multiple significant predictors, we strategically developed our risk stratification model using two immediately available parameters: age and initial rhythm. This simplified approach offers practical advantages in acute clinical settings where rapid decision-making is crucial. To quantify the discriminative trade-off, we compared the C-statistic across three models: the full multivariate model (AUC = 0.812), the bedside model using low-flow time, age, and initial rhythm (AUC = 0.751), and the simplified stratification (AUC = 0.645). The AUC difference of 0.054 (bootstrap 95% CI: 0.011–0.099) supports the pragmatic trade-off. A sensitivity analysis restricted to IHCA patients (*n* = 236) demonstrated comparable performance (AUC = 0.818), confirming robustness. Unlike more complex scoring systems that require multiple laboratory values or detailed clinical parameters, these two factors can be immediately evaluated upon patient presentation, enabling quick risk stratification and appropriate low-flow time guidance. This approach represents a significant advancement over previous studies that either provided uniform cutoff times or utilized complex scoring systems. While previous risk assessment models have incorporated multiple variables requiring laboratory results or detailed clinical assessments, our streamlined approach maintains predictive power while offering immediate clinical applicability. This more nuanced understanding of the time-outcome relationship could help clinicians make more informed decisions about ECPR continuation or termination based on individual patient risk profiles.

The age-dependent variation in ECPR outcomes observed in our study necessitates careful examination of underlying physiological mechanisms. Our analysis identified age as a significant prognostic factor, demonstrating a marked increase in poor outcomes around age 50. Several physiological factors explain this age-related threshold effect. Younger patients characteristically demonstrate greater cardiovascular reserve, more robust microvascular function, and enhanced cellular resilience to ischemia–reperfusion injury. These inherent physiological advantages enable them to better tolerate prolonged periods of CPR without developing irreversible end-organ damage. Our findings align with previous studies that have documented age-related differences in CPR outcomes, consistently showing better survival rates and neurological outcomes in younger patients.

The initial cardiac rhythm emerged as another crucial determinant of ECPR outcomes in our study, with shockable rhythms demonstrating significantly better neurological outcomes compared to non-shockable rhythms. Our risk-stratified analysis revealed that patients with initial shockable rhythms could tolerate longer low-flow times while maintaining the possibility of favorable outcomes. This observation aligns with previous studies demonstrating higher survival rates in ECPR patients with initial shockable rhythms. The pathophysiological basis for this difference likely involves better preserved cardiac mechanical function and reduced metabolic derangement in cases of shockable rhythms. Furthermore, these patients often have more reversible underlying causes and may benefit from concurrent interventions such as coronary revascularization during ECPR support. The enhanced recovery potential in shockable rhythms may also be attributed to less severe ischemia–reperfusion injury, as these patients typically receive earlier defibrillation attempts and maintain better coronary perfusion pressures during resuscitation efforts. Our findings are consistent with recent systematic reviews confirming that younger age, shockable rhythm, and shorter low-flow time are robust predictors of favorable ECPR outcomes [22,23,24]. Yu et al. demonstrated that the interplay between age and low-flow duration significantly influences neurological outcomes [5]. A large nationwide study further showed that the effective ECPR time window is significantly shorter in elderly patients [25]. The landmark ARREST trial demonstrated that early ECMO-facilitated resuscitation significantly improved survival to hospital discharge compared with standard ACLS in patients with refractory ventricular fibrillation, providing the first randomized evidence supporting ECPR in selected populations [26]. An updated meta-analysis including both randomized trials and propensity-matched studies further confirmed that ECPR reduces in-hospital mortality (OR 0.63, 95% CI 0.50–0.79) and improves neurological outcomes, with particularly notable benefits in IHCA settings [27]. Our analytical approach intentionally avoided fixed sensitivity/specificity-based cutoffs; presenting the full spectrum of observed outcome rates allows clinicians to make contextually appropriate decisions [28]. In this context, the Pre-ECPR score developed by Redfors et al. similarly integrated multiple weighted predictors as continuous variables rather than arbitrary cutoffs, demonstrating significantly better discriminatory performance than ELSO criteria alone, further supporting the value of multivariable approaches to ECPR patient selection [29].

Our study has several limitations. The low-risk group (age <50 years with shockable rhythm) comprised only 20 patients; consequently, the observed low-flow time thresholds in this subgroup should be considered exploratory findings. In the high-risk group, the modest discriminative ability of low-flow time (AUC = 0.624) suggests that the outcome is driven by factors beyond resuscitation duration. For OHCA patients (15.4%), low-flow time was calculated from EMS dispatch time, which may not precisely reflect true arrest onset. As a single-center retrospective study, the findings may be subject to selection bias. During the eight-year study period, evolution in ECPR techniques, equipment, and protocols, along with improving team experience, may have influenced outcomes. The generalizability of our results requires careful consideration, as our study was conducted at a tertiary care center with an established ECPR program and experienced ECMO team. Our findings might not be directly applicable to centers with different resource levels or ECPR experience. Furthermore, missing data and the inherent subjectivity of CPC scoring at hospital discharge may affect the robustness of our conclusions. Multi-center prospective studies with longer follow-up periods will be necessary to validate our findings. Nevertheless, our study provides valuable insights into optimizing low-flow time assessment based on patient risk stratification.

## 5. Conclusions

In conclusion, this study provides evidence that the prognostic significance of low-flow time (cardiac arrest to ECMO pump-on) should be evaluated based on stratified risk groups rather than applying uniform criteria to all patients. Our findings demonstrate that the acceptable low-flow time for achieving favorable neurological outcomes varies substantially among different risk groups, with low-risk patients (younger age with shockable rhythms) showing potential for good neurological recovery even with extended low-flow times of up to 70 min. This risk-stratified approach may enable more nuanced clinical decision-making in ECPR implementation, potentially improving patient selection and timing of ECPR termination. Further prospective multicenter studies are warranted to validate these findings and establish their generalizability across different clinical settings.

## Figures and Tables

**Table 1:** Baseline characteristics of patients.

	Favorable Neurologic Outcome (*n* = 120)	Poor Neurologic Outcome (*n* = 159)	*p* Value
Patient demographics			
Age, years	56.0 ± 17.6	59.7 ± 17.6	0.084
Sex, male	85 (70.8)	115 (72.3)	0.999
Comorbidities			
Hypertension	50 (41.7)	82 (51.6)	0.129
Diabetes mellitus	34 (28.3)	57 (35.8)	0.231
Cardiovascular disease	23 (19.2)	37 (23.3)	0.497
Malignancy	16 (13.3)	31 (19.5)	0.230
Chronic kidney disease ^a^	7 (5.8)	24 (15.1)	0.025
Stroke	10 (8.3)	9 (5.7)	0.524
CPR details			
Type of cardiac arrest			0.045
Out-of-hospital cardiac arrest	12 (10.0)	31 (19.5)	
In-hospital cardiac arrest	108 (90.0)	128 (80.5)	
First monitored rhythm			0.003
Asystole	13 (10.8)	35 (22.0)	
Pulseless electrical activity	60 (50.0)	88 (55.3)	
Shockable rhythm (VT or VF)	47 (39.2)	36 (22.6)	
Low-flow time (cardiac arrest to ECMO pump-on), minutes	27.1 ± 18.1	39.5 ± 22.8	<0.001
Cause of CPR			0.001
Cardiac cause	88 (73.3)	85 (53.5)	0.003
Ischemic cardiac disease	69 (57.5)	62 (39.0)	
Non-ischemic cardiac cause	19 (15.8)	23 (14.5)	
Non-cardiac cause	32 (26.7)	74 (46.5)	
Location of ECPR			0.999
Intensive care unit	37 (30.8)	74 (46.5)	
Emergency department	26 (21.7)	49 (30.8)	
Coronary catheterization lab	46 (38.3)	26 (16.4)	
Operation room	11 (9.2)	7 (4.4)	
General ward	0 (0)	3 (1.9)	
Management in the intensive care unit			
Targeted temperature management ^b^	12 (10.0)	28 (17.6)	0.105
Continuous renal replacement therapy	36 (30.0)	80 (50.3)	0.001
Intra-aortic balloon pump	4 (3.3)	6 (3.8)	0.999
Complication during ECMO			
ECMO site bleeding	15 (12.5)	21 (13.2)	0.999
Limb ischemia	7 (5.8)	13 (8.2)	0.605
Stroke after ECPR	5 (4.2)	9 (5.7)	0.773
Rhabdomyolysis	6 (5.0)	4 (2.5)	0.435
Gastrointestinal bleeding	1 (0.8)	7 (4.4)	0.160
Sepsis	0 (0)	6 (3.8)	0.083
Laboratory data			
Hemoglobin, g/dL	10.5 ± 2.6	9.3 ± 3.1	0.001
Platelet, ×10^3^/μL	203.5 ± 109.6	182.2 ± 101.4	0.103
Total bilirubin, mg/dL	1.2 ± 1.6	1.5 ± 2.2	0.091
Aspartate transaminase, U/L	145.7 ± 341.4	311.5 ± 804.6	0.025
Alanine transaminase, U/L	108.0 ± 248.1	158.2 ± 310.0	0.145
Blood urea nitrogen, mg/dL	20.3 ± 13.6	28.3 ± 19.2	<0.001
Creatinine, mg/dL	1.4 ± 1.3	2.1 ± 2.2	0.001
Troponin I, μg/L	18.2 ± 69.5	24.9 ± 71.0	0.502
HCO3^-^, mmol/L	15.7 ± 4.6	16.1 ± 6.1	0.589
Initial lactate, mmol/L	9.5 ± 5.2	12.5 ± 4.7	<0.001

^a^ Chronic kidney disease is defined as either kidney damage or a glomerular filtration rate less than 60 mL/min/1.73 m^2^ for 3 months or longer. ^b^ Targeted temperature management was performed by using a surface cooling device (Arctic Sun Temperature Management System; BD, Becton, Dickinson and Company, Franklin Lakes, NJ, USA). Data are presented as numbers (%) or means ± standard deviations. CPR, cardiopulmonary resuscitation; VT, ventricular tachycardia; VF, ventricular fibrillation; ECPR, extracorporeal cardiopulmonary resuscitation; ECMO, extracorporeal membrane oxygenation.

**Table 2:** Multivariable analysis of factors associated with poor neurological outcomes.

	Adjusted OR (95% CI)	*p*-Value
Age greater than 50 years and asystole	4.89 (1.41–17.00)	0.012
Age greater than 50 years and pulseless electrical activity	9.70 (2.80–33.60)	<0.001
Aspartate transaminase, U/L ^a^	1.52 (1.15–1.99)	0.003
Creatinine, mg/dL ^a^	2.08 (1.30–3.34)	0.002
Initial lactate, mmol/L ^a^	1.88 (1.27–3.45)	0.041
Low-flow time, min ^a^	3.50 (2.02–6.06)	<0.001

^a^ Data were log-transformed to reduce skewness. OR, odds ratio; CI, confidence interval; ECPR, extracorporeal cardiopulmonary resuscitation.

**Table 3:** Relationship between low-flow time and the cumulative probability of favorable neurological outcomes.

Low-Flow Time of ECPR (min)	All Patients (*n* = 279)	Low Risk (*n* = 20)	Moderate Risk (*n* = 110)	High Risk (*n* = 149)
10	16 (13.3)	1 (7.7)	6 (10.9)	9 (17.3)
20	50 (41.7)	3 (23.1)	22 (40.0)	25 (48.1)
30	79 (65.8)	6 (46.2)	37 (67.3)	36 (69.2)
40	100 (83.3)	8 (61.5)	47 (85.5)	45 (86.5)
50	108 (90.0)	10 (76.9)	50 (90.9)	48 (92.3)
60	112 (93.3)	12 (92.3)	52 (94.5)	48 (92.3)
70	116 (96.7)	13 (100)	53 (96.4)	50 (96.2)
80	117 (97.5)	13 (100)	54 (98.2)	50 (96.2)
90	120 (100)	13 (100)	55 (100)	52 (100)
100	120 (100)	13 (100)	55 (100)	52 (100)

Values are reported as cumulative number of patients (%).

**Table 4:** Reference cutoff for low-flow time stratified by risk group.

	Cutoff Time (min)	Sensitivity	Specificity	AUC (95% CI)
Low-risk	38	0.857	0.462	0.700 (0.457–0.881)
Moderate-risk	27	0.800	0.382	0.645 (0.549–0.734)
High-risk	20	0.835	0.462	0.624 (0.541–0.702)

AUC, area under the curve; CI, confidence interval.

## Data Availability

Regarding data availability, our data are available on the Harvard Dataverse Network. The data supporting the findings of this study are openly available on the Harvard Dataverse Network: https://doi.org/10.7910/DVN/BCVRSH (accessed on 25 June 2025).

## Abbreviations

CPC	Cerebral Performance Categories
CPR	conventional cardiopulmonary resuscitation
ECMO	extracorporeal membrane oxygenation
ECPR	extracorporeal cardiopulmonary resuscitation
VF	ventricular fibrillation
VT	ventricular tachycardia
